# Two Methods for Engaging with the Community in Setting Priorities for Child Health Research: Who Engages?

**DOI:** 10.1371/journal.pone.0125969

**Published:** 2015-05-04

**Authors:** Wavne Rikkers, Katrina Boterhoven de Haan, David Lawrence, Anne McKenzie, Kirsten Hancock, Hayley Haines, Daniel Christensen, Stephen R. Zubrick

**Affiliations:** 1 Telethon Kids Institute, The University of Western Australia, Perth, Western Australia, Australia; 2 School of Population Health, The University of Western Australia, Nedlands, Perth, Australia; Centre for Geographic Medicine Research Coast, KENYA

## Abstract

**Objective:**

The aims of this study were to assess participatory methods for obtaining community views on child health research.

**Background:**

Community participation in research is recognised as an important part of the research process; however, there has been inconsistency in its implementation and application in Australia. The Western Australian Telethon Kids Institute Participation Program employs a range of methods for fostering active involvement of community members in its research. These include public discussion forums, called Community Conversations. While participation levels are good, the attendees represent only a sub-section of the Western Australian population. Therefore, we conducted a telephone survey of randomly selected households to evaluate its effectiveness in eliciting views from a broader cross-section of the community about our research agenda and community participation in research, and whether the participants would be representative of the general population. We also conducted two Conversations, comparing the survey as a recruitment tool and normal methods using the Participation Program.

**Results:**

While the telephone survey was a good method for eliciting community views about research, there were marked differences in the profile of study participants compared to the general population (e.g. 78% vs 50% females). With a 26% response rate, the telephone survey was also more expensive than a Community Conversation. The cold calling approach proved an unsuccessful recruitment method, with only two out of a possible 816 telephone respondents attending a Conversation.

**Conclusion:**

While the results showed that both of the methods produced useful input for our research program, we could not conclude that either method gained input that was representative of the entire community. The Conversations were relatively low-cost and provided more in-depth information about one subject, whereas the telephone survey provided information across a greater range of subjects, and allowed more quantitative analysis.

## Introduction

Consumer and community participation in research is now recognised in Australia as an important and integral part of the research process by research funding bodies such as the National Health and Medical Research Council (NHMRC). [1] However, not all researchers incorporate consumer and community participation into their research, either as a result of not valuing this type of input or because they do not understand the various methods by which community members can participate. In addition, not all members of the public are aware of the opportunities which exist for them to contribute to a process that might be seen as being outside their sphere of influence or expertise. To date there has been limited research in Australia targeted at the comparative benefits and applicability of different methods for gaining community participation or in understanding more about the types of people willing to participate in research. This study seeks to address this gap by focussing on the characteristics and views of participants involved in two participatory methods.

The term ‘consumer and community participation’ can be interchangeable with consumer and community engagement or involvement. [2] For the purposes of this study, the terms ‘participation’ or ‘involvement’ exclude participation as a subject in a research study. We defined consumers as people who directly or indirectly make use of a service. They may include patients, carers, organisations representing consumers’ interests, or members of the public. Community members are defined as a group of people sharing a common interest but not necessarily a common geographic location. A person or consumer may belong to more than one community and contribute to different community views. [3] Public deliberation or deliberative processes can take several forms and be described in many ways. For the purpose of this study, they are interpreted as processes which involve members of the public in helping to inform or to make decisions about issues affecting them. [4]

The study reported in this paper specifically relates to the work of one research group at the Telethon Kids Institute (the Institute) whose overall research area is broadly classified as *human capability expansion*. In general terms, this field examines how families and communities can make the best use of the resources available to them to maximise the potential of their children to develop into healthy, well-adjusted adults who are able to live productive, contributing lives that they choose and value.[5] As such, the group currently has active research interests in several areas that are important in healthy child development, including mental health, education, language development, nutrition and obesity. When determining priorities for future research work between these areas, and determining detailed priorities within each area, the most appropriate consumer group is considered to be the whole community.

There are many possible ways of involving the community in research, which include all phases of the research cycle, such as, deciding what to research, how to conduct the research, and disseminating and translating the results. [6,7,8] As a matter of principle, the case for consumer and community involvement in research is straight-forward. The vast majority of public health research in Australia, as in other countries, is funded by the community, either through government appropriations such as via the NHMRC, direct government funding to Universities and research institutions, or through charitable organisations.

As there are more research ideas proposed in Australia than there are funds available to support [9,10], it is important that decisions about the relative priority to be given to different fields of research endeavour should align with the challenges facing Australian society. However, processes for community members to provide input into decision making about priorities for research and its funding appear to be limited and ad hoc in Australia.

Since the 1980s, a strong movement developed in Australia recognising that consumers should be meaningfully involved in decision making about their health care and treatment, as well as broader health policy, planning and service delivery.[11] The wide adoption of consumer and community input in the health research sector 20 years later [12] may have been facilitated by the already strong network established for consumer participation in health care service delivery. NHMRC released their *Statement on Consumer and Community Participation in Health and Medical Research* in 2002, developed in partnership with the Consumers Health Forum of Australia (CHF). [13,14] This was followed by the release of a participation framework and resource pack in 2005. [15,16] While consumer and community participation has been included in a wider range of health services research, its uptake has been much slower in basic research and observational studies that do not include interventions. [2,17,18]

Although there are several documents and websites which support and provide processes for participation in research by members of the community, a review of the Australian literature did not reveal many examples of investigations into different methods for consumer and community participation in research per se. Apart from recent papers by Saunders and Girgis [19,20] into consumer involvement in health and medical research, there appears to be little relevant Australian research about this subject. Internationally, there is a significant body of literature regarding public deliberation processes, most of which focus on health services, policy or resource allocation, and more recently, in the field of genomics research. [21, 22, 23, 24] However, there is a need for more investigation into how deliberative processes compare with other methods and which method is better suited to public engagement in priority setting. [25]

In Western Australia (WA), the Telethon Kids Institute’s Participation Program is underpinned by its Consumer and Community Participation Policy, which promotes the establishment and building of relationships between the researchers at the Telethon Kids Institute and consumers and community members. [26] This policy includes a number of methods for fostering active involvement of community members in various stages of research.[27,28] One such method discussed in this paper is that of consumer and community consultation forums known as Community Conversations. Developed in collaboration with the Consumer and Community Advisory Council [29], these forums are similar in nature to the public deliberation process adopted in other countries [30,31], although they differ in that all opinions are collected and valued, that is, there is little deliberative work done to determine one or the ‘right’ answer or decision. Involving one-off meetings between consumers, community members and researchers, they are conducted using the world café methodology, which is a facilitated collaborative dialogue to encourage the sharing of knowledge and ideas in a way that identifies priorities, future actions, and provides feedback and input into specific issues, topics and research findings.[6,32] Attendees are generally recruited through a Participation Network run as part of the Participation Program, and consumers and community members are encouraged through an informal environment to express opinions based on their lived experiences or fields of interest. This type of phenomenological approach is useful for researchers to gain perspectives into research topics and into ideas for further research that might not otherwise be apparent from data analysis alone or considered by researchers, clinicians, funding bodies or government agencies. [33]

While the Participation Program has a very positive and encouraging level of participation for its Community Conversations (40 events with 1300 participants to date), it is recognised that this number represents only a small sub-section of the population of Western Australia. Usually, each Community Conversation is focussed on a single research area or health condition, attended by 20–40 participants interested in that particular subject. However, the *human capability expansion* research area encompasses a wide research agenda, applicable to a large part of the community, and therefore we sought to test a telephone survey as another method to canvas the views of a wider spread of the population.

Telephone surveys are widely used in public opinion polling and market research. In addition, they have also played a vital role in public health practice and research through the monitoring of risk factors and behaviours, and prevalence of health conditions, as well as assessing efficacy of interventions. [34] Sampling techniques using random digit dialling of landlines have proved useful for large population based surveys. However, research has also shown that there are drawbacks associated with this methodology, including possible coverage bias and restricted generalisability of results related to the emerging use of new communication technology such as cell phones and tablets.

Keeping these restrictions in mind, we wanted to see if a wider range of community views on priorities for our research agenda could be obtained from a telephone survey approach, without the accompaniment of the edifying information that is typically provided during a Community Conversation. We also wanted to capture views on community participation in the research process; and given the time and effort expended in recruiting people to attend a Community Conversation, we wished to determine the feasibility of recruiting people to attend a Conversation using a ‘cold calling’ telephone approach and to compare their views and characteristics with those of people recruited via the Participation Network. In a further effort to reduce costs, we also planned to select respondents without the benefit of quota sampling and to measure how representative of the general population the resulting survey population might be. Lastly, a key feature of the Conversations is that many attendees have a lived experience of the issue being discussed, so we wanted to use the telephone survey to explore how lived experience of a condition can influence a person’s views.

## Materials and Methods

This study was approved by the Human Research Ethics Committee of The University of Western Australia (reference no. RA/4/1/6244). In the interests of reducing respondent burden as well as survey costs, verbal rather than written consent was used for the telephone survey. All respondents were informed that the telephone survey was voluntary and that they were free to withdraw at any time. Once verbal consent was given, this was recorded on the survey form and survey participants were assured that no identifying information about them would be released as per Federal privacy laws. Similarly, participants at the Community Conversations were given the same assurances. They received written invitations to attend and their written acceptance of the invitation was taken as consent. The Human Research Ethics Committee of the University of Western Australia approved this consent procedure.

### Methods for capturing community views

Our study involved the administration of a voluntary telephone survey to two groups of people. The first group comprised people selected using random digit dialling of landlines in Western Australia. The second comprised people who first attended a Community Conversation by invitation through the Participation Program. In addition, we compared two different means of recruiting participants to attend Community Conversations, that is, the telephone survey and the Participation Program.

### Telephone Survey

We conducted a 15 minute telephone survey which included questions on a range of *human capability expansion* and public health research areas, people’s firsthand experience of issues or conditions (lived experience), opinions about community participation in research and general demographic information.

The sample was chosen using random digit dialling of landlines to select 800 households across Western Australia, with calls being made during the hours of 5:00pm to 8:30pm on weekdays and 10:00am to 5:00pm on Saturdays and Sundays. Some call backs were made during the day to people too busy to speak during the evening. Households without a landline were excluded from the scope of the survey, which equated to a sample loss of approximately 23% of WA households [35] or approximately 19% of the 18+ year old population. [36] Likert Scales were used to measure people’s opinions, with respondents asked to provide a rating from 0 to 10, where 0 equated to the lowest measure and 10 to the highest, for example, ‘extremely unimportant’ and ‘extremely important’. The survey questionnaire is included in S1 Appendix.

### Community Conversations

In order to identify differences in views that might be related to different recruitment methods, two Community Conversations were conducted at the Telethon Kids Institute on the topic of childhood education and with the aim to include up to 30 participants for each. The two Conversations were conducted with an identical format, on consecutive evenings.

The attendees for the first Community Conversation were recruited through the Participation Program using their traditional method of inviting people from the Participation Network, as well as promotion on www.involvingpeopleinresearch.org.au and through other consumer and community organisations, which were relevant to the topic being discussed (childhood education). The attendees for the second Community Conversation were recruited by means of an invitation proffered during the telephone survey. If they responded positively, at the end of the survey they were invited to attend the Community Conversation.

Attendees at both Community Conversations were given the same presentation [37] on childhood education research into patterns of attendance and their effect on academic performance as measured by the National Assessment Program—Literacy and Numeracy. [38] The education-related topic was chosen as one which would have wide appeal, would be considered relatively non-controversial and represented a current *human capability expansion* research topic. The presentation was followed by facilitated small group discussions of reactions to the results, solutions on how to overcome issues identified in the results, and methods for how results could be fed back to the community. Additionally, attendees were also asked to identify priorities for future research around the general topic of education. The questions used to stimulate discussion are included in S2 Appendix.

## Results

### Telephone Survey Results

The survey had a response rate of 26%, yielding 816 respondents in total, 10 of whom were also participants in a Community Conversation. Of these 10 respondents, two respondents undertook the survey first and were subsequently recruited to attend a Community Conversation. The other eight were recruited to attend a Conversation via the Participation Program and were administered the telephone survey in the week following the Community Conversations. Due to the small number of attendees at the Conversations, it was not possible to use their results in a statistically meaningful way.

#### General opinions on child health research

**Fig 1:**
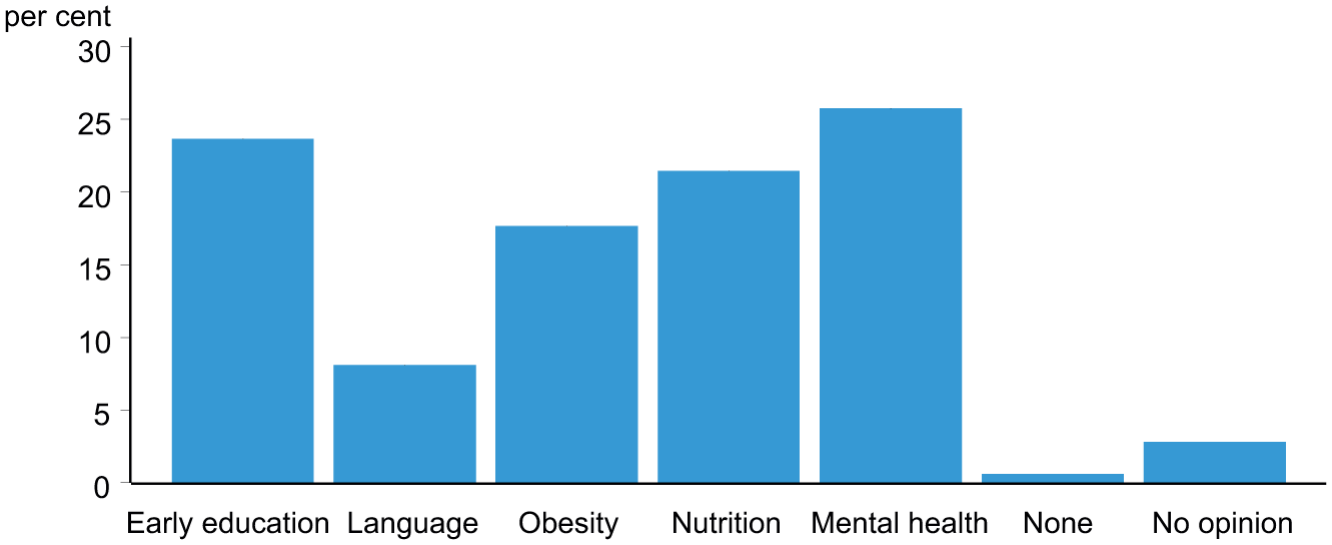
Most important research area. Respondents were asked to rate the importance of the *human capability expansion* research areas—early childhood education, language development, childhood obesity, children’s nutrition, and children’s mental health.

Respondents were asked their views about the importance of several *human capability expansion* research areas: early childhood education, language development, childhood obesity, children’s nutrition, and children’s mental health. Children’s mental health was chosen as the most important area to research (Fig 1) with nearly 26% of people choosing this option compared with 8% for language development.

#### Community participation in research

When asked how important it was for community members to have a say in the different stages of the Institute’s research process, higher ratings were given to the importance of setting research priorities, planning the research and the use of the research results than to how to conduct the research. Also, the majority of respondents (73%) ranked the Institute researchers ahead of both the government and members of the community in terms of who should have the most say in determining the Institute’s research priorities.

We sought people’s views on the most effective methods for the community to provide input into research. People willing to attend a Community Conversation gave the highest ratings of importance to participating in focus groups or workshops (8.1 out of 10) or being represented on advisory groups (8 out of 10). Others gave their highest ratings (7.5 out of 10) to donating money to specific areas of research and being represented on advisory groups.

#### General profile of telephone survey respondents

Of those who completed the telephone survey, 587 (72%) were female and 229 (28%) were male, compared with 50% of each gender in the general population.[39] Although the greatest proportion (28%) of participants fell in the age range of 35–44 years, 23% of people were aged 65 years or more, compared with 18% of the general population. Ninety two per cent (750) of the respondents had children, and of these, 475 had children currently living with them at the time of the survey. Sixty five per cent of respondents had school-aged children. We were unable to find reliable comparative data on the number of parents in Western Australia. A total of 570 people (70%) were married, compared with 49% of the general population, and only 47% of respondents were currently in paid employment, compared with nearly 60% of the general population. Nearly 57% of survey respondents had completed a tertiary qualification compared with 42% of the general population. See Tables 1 and 2 for a detailed comparison of the survey population with the general population.

**Table 1:** Demographic characteristics of telephone survey population compared with Western Australian population.

Characteristic		Survey Sample	WA Population^a^
		%	%
Marital status	Single	6.6	35.3
	Married	69.9	48.7
	In a de facto relationship^b^	9.4	-
	Separated	2.4	3.0
	Divorced	4.7	8.4
	Widowed	5.6	4.6
	Prefer not to say	1.4	-
Gender	Male	28.1	50.3
	Female	71.9	49.7
Age	18–24 yrs	2.0	12.0
	25–34 yrs	12.9	18.5
	35–44 yrs	27.8	18.4
	45–54 yrs	20.7	17.9
	55–64 yrs	13.5	14.9
	65+ yrs	23.0	18.2
	Prefer not to say	0.1	-
Highest level of education	Tertiary qualification	57.6	49.6c
	Year 11–12	26.2	26.9
	Year 8–10	13.5	22.4
	No formal education	0.5	-
	Prefer not to say	2.2	-
In paid employment^d^	Yes	46.8	59.9
	No	50.5	40.1
	Prefer not to say	2.7	
Country of birth	Australia	69.5	62.9
Income	No income	0.7	1.3
	Less than $32,000 pa	16.2	17.2
	$32,000 < $65,000 pa	13.7	20.9
	$65,000 <$130,000 pa	22.7	26.2
	$130,000 <$210,000 pa	11.4	18.0
	$210,000 or more pa	5.3	4.2
	Prefer not to say	30.0	12.3

^a^ Australian Bureau of Statistics: 2011 Census of Population and Housing http://www.censusdata.abs.gov.au/census_services/getproduct/census/2011/quickstat/5?opendocument&navpos=220

^b^ Marital status classification for 2011 Census of Population and Housing, Australia does not include De facto couples

^c^ Australian Bureau of Statistics *Education and Work*, *Australia*, *May 2014*, *cat*.*no*. *6227*.*0*. Canberra: ABS, 2014. Available at http://www.abs.gov.au/AUSSTATS/abs@.nsf/DetailsPage/6227.0May%202014?OpenDocument

^d^ These figures were calculated using the whole population 18 years and over, not just those in the Labour Force.

**Table 2:** SEIFA Index of relative socio-economic disadvantage based on postcode of residence, 2011 Census of Population and Housing.

SEIFA disadvantage	Survey Sample (%)	WA Population (%)
Not stated	4.5	-
Bottom quintile	7.7	10.2
2^nd^	20.8	20.2
3^rd^	16.0	21.4
4^th^	20.8	16.4
Top quintile	30.0	31.8

Distribution of telephone survey population compared with WA population.

#### Involvement in community activities

Respondents were asked if they had participated in a range of community activities in the previous 12 months, such as belonging to a special interest action group, a school Parents & Citizens association or doing volunteer work for a group or organisation. Table 3 shows that volunteering time was the most participated in community activity (48.8%). In comparison, nine out of the ten people who attended a Community Conversation had done volunteer work.

**Table 3:** Involvement in community activities.

		Community activities			
		Consumer advocacy or special interest action group	Local school parents & citizens assoc.	Local community forum	Volunteering time or services for a group or organisation
		Mean%	Mean%	Mean%	Mean%
Participation in activity	No	91.7	76.7	78.9	51.2
	Yes	8.3	23.3	21.1	48.8

#### Lived experience

Respondents were asked a series of questions to determine if and how lived experience of an issue influenced their opinions about that issue. For those with experience of mental health problems and mental health services, there was a slightly higher preference for research into the treatment rather than the causes of mental health problems in children. (Table 4)

**Table 4:** Whether more important to research causes or treatment of mental health problems by lived experience of mental health problem or services.

		Most important aspect to research in mental health problems in children				
		Causes	Treatment	Equally important	Neither	No opinion
		%	%	%	%	%
**All survey respondents**		57.1	23.0	17.4	0.6	1.8
**Experience of a mental health problem**	Yes	51.9	25.8	20.8	0.5	0.8
	No	61.5	20.5	14.5	0.6	2.6
	Prefer not to say	44.4	33.3	22.2	-	-
**Experience of mental health services in WA**	Yes	49.8	28.2	20.9	0.6	0.3
	No	61.8	19.5	15.0	0.6	2.8
	Prefer not to say	50.0	50.0	-	-	-
	Unsure	57.1	21.4	21.4	-	-

Respondents were asked to choose whether everyone or only those with lived experience of a condition should have the most say in determining research priorities into that condition. Sixty per cent of all respondents chose those with the lived experience of the condition. Table 5 shows a comparison of responses according to whether the respondent had a lived experience of a serious health problem. There was little difference in the patterns of response. When asked if they or any member of their immediate family had a serious health problem (i.e. an indicator of lived experience of health issues), 30% of all survey participants responded that they had.

**Table 5:** Who should have most say in determining research priorities, by whether respondent has experience of a serious health problem.

		Experience of serious health problem		
		No	Yes	Prefer not to say
		%	%	%
**Who should have a say**	Everyone	17.3	20.3	14.3
	Only those with experience of a condition	60.1	57.0	28.6
	Both	17.0	14.3	14.3
	Neither	1.2	1.8	28.6
	Don’t know	1.2	0.9	-
	No opinion	0.4	1.6	-

#### Participation in the Community Conversations

The first Community Conversation, for which attendees were recruited through the Participation Program, was attended by 11 people, which was lower than anticipated—it was hoped 20–30 people would attend. Three of the participants identified as being members of organisations involved in providing educational services and elected not to participate in the telephone survey administered in the week following the Conversation.

During the telephone survey, 167 people indicated they would be interested in attending a Community Conversation generally, with 39 of those people willing to receive a formal invitation to a specific Community Conversation on childhood education research. Twenty survey respondents accepted the invitation, however, only three people attended (two respondents plus one partner). The low number of attendees prohibited meaningful analysis of the two conversations.

The demographic profile of the attendees differed markedly from the general population. See Table 6.

**Table 6:** Demographic characteristics of Community Conversation attendees, compared with WA Population.

Characteristic		Attendees	WA Population
		%	%
Marital status	Single	12.5	35.3
	Married	87.5	48.7
Gender	Male	28.3	50.3
	Female	71.6	49.7
Highest level of education	Tertiary qualification	87.5	30.9
	Year 11–12	12.5	44.8
In paid employment	Yes	62.5	59.9
	No	37.5	40.1
Annual household income before tax	$32,000–<$65,000	25.0	20.9
	$65,000–<$130,000	12.5	26.2
	$130,000–<$210,000	12.5	18.0
	$210,000 or more	50.0	4.2
Country of birth	Australia	62.5	62.9

Research priorities identified during the Community Conversations are listed in more detail in S2 Appendix.

## Discussion

This study demonstrated that a telephone survey could provide useful information about priorities for a broad child health research agenda and community participation in research when the respondents were provided with no edifying information. However, we found that it was not a feasible method for recruiting people to attend a Community Conversation, and the resulting number of attendees was too small to allow for any quantitative analysis to be performed, with only two (plus one partner) out of 816 people in the survey attending. While the survey response rate of 26% allowed us to secure 816 respondents within our budget, we found that the survey population was not representative of the general WA population. A much larger budget would be needed to achieve this outcome though the use of quota sampling. On the positive side, the sample size provided opportunity for quantitative analysis of responses and the 15 minute survey duration allowed for the inclusion of a wide range of inter-related topics and questions about child health research and community involvement in research. Lastly, there were some, albeit small, differences in the views of those with a lived experience of a condition when compared with the views of others, in that a greater proportion of them preferred research into treatments or services over causal research.

We found that the telephone survey elicited a range of useful views to inform our future research priorities and on how we should involve the community in our research program. Respondents were comfortable with assigning priorities and values to different child health research focus areas without the need for more clarification about our research or the Institute. All areas of *human capability expansion* research were strongly endorsed by most respondents, with little variation in their scores.

Respondents were almost uniform in saying that it was important for the community to “have a say” in setting the Telethon Kids Institute’s research priorities. Yet while they placed a high level of importance on helping researchers decide on the most important things to research, planning the research and on how to use the results, less importance was attributed to the community having a say in planning aspects of the research itself. They also placed Institute researchers ahead of community members or the government in terms of who should decide what research projects are undertaken by the Institute. From the survey results, one could conclude that the community see less of a role for themselves in the conduct of research and that this stage of the process falls mostly into the realm of the researcher. It is possible that many consumers and community members are not aware of all the ways in which they can be involved in or contribute to research. They may also not be aware of, or may not be offered opportunities to contribute to, research projects. Therefore, it is important for organisations wishing to involve lay people in their research processes to provide avenues for involvement, training and support to enable useful input from confident, capable consumer and community members. [40]

When asked about the effectiveness of different methods for the community to help shape decisions regarding the Telethon Kids Institute’s research, there were slight differences in opinion between those willing in principle to attend a Community Conversation and those not. Not surprisingly, those willing to attend gave the highest rating to participating in forums or workshops, while those not willing to attend chose donating money to specific areas of research or having a community representative on advisory groups as the most effective methods.

We had also aimed to see how closely the telephone survey population profile resembled that of the general population, without benefit of quota sampling. Neither of the participation methods (the phone survey and the Community Conversations) secured participation from groups of people who were statistically representative of those living in WA. Study participants were significantly at variance with the general adult WA population across several variables including gender, age, marital status, and education.

Survey participants also had a higher level of volunteering than the general population (49% compared with 38%). [41] Quite possibly, respondents were partially motivated to participate in the survey because of a stronger ‘social conscience’ and a desire to interact with the community in a contributive way. The ABS General Social Survey 2010 showed that people who volunteer are more likely to be involved in other aspects of community life. [42] Notably, of those who attended a Community Conversation, 90% had volunteered their services in the previous 12 months.

There are a number of possible explanations for the survey population’s lack of representativeness of the general population. The telephone survey excluded households without a landline, which increased the risk of possible coverage bias. [43,44] According to the Australian Communications and Media Authority, in 2012 approximately 3.3 million Australians lived in mobile-only households. [36] Studies both locally and abroad have shown these households typically include people below the age of 35 years, single, males, students, renters, highly mobile people and low income earners [35,43,44], most of whom were under-represented in our study population.

It is also highly possible that the topic of the survey influenced people’s participation. While the main aims of the study focussed on the types of people willing to be involved in a part of the research process, an open question prior to the project was whether people selected at random to participate in a telephone survey would have, and be willing to express, opinions on priorities in child health research. We found that most people participating in the telephone survey were highly interested in the topic of child health research and were willing to express an opinion or to rate priorities for a range of research topics. According to Bosnjak and Batinic, participation in surveys is motivated by the respondent’s perception of the social importance of the survey topic, its seriousness and its relevance to the respondent. [45] Clearly, the profile of the respondent population (92% parents) indicates that the subject of child health was one which fit these criteria. Although the majority of respondents (65%) had school-aged children, it is interesting to note that some grandparents and parents of adult children were also motivated to participate in the survey.

A common criticism of many methods for community engagement in research, and indeed most public deliberative processes, is that they lack representativeness and that the priorities described by the few may not represent those of the many. [46] However, Goold et al posit that representativeness does not need to be limited to demographic characteristics but could include lived experiences or health conditions for example. [47] This begs the question of whether our respondents may in fact be considered representative of those interested in aspects of child health since the vast majority of them were parents. However, other studies of community involvement in research suggest that capturing a diversity of views is as important as representativeness. [48] As such, our inability to capture views from sufficient single young adults, males, or low income earners may be considered inadequate, and as advised by Abelson et al (2003), it may be contingent on us as researchers to be more creative in how we include these groups in future community engagement exercises. [49] However, the relatively low response rate combined with the skewed demographic profile of those willing to participate indicates that a much larger economic outlay would be required to achieve a truly representative sample.

Relevant to a possible link between the large number of parents in the survey and the topic of child health, was the inclusion of questions designed to allow analysis of people’s views according to whether or not they had a lived experience of a research issue. People’s lived experience is gained through their first-hand knowledge of a particular health condition or issue as a consumer or carer, or through social circumstance or environment. It can add a real-life perspective to views expressed regarding research into those conditions or issues and is often associated positively with those participating in deliberative processes or public discussion forums. [4,7,46,48,49,50] However, some researchers have criticised the subjective nature of lived experience and question its value in providing input to a scientific research process. [17] In consideration of this criticism, this study allowed us to examine how lived experience might shape people’s views with respect to assigning research priorities and to gain a deeper understanding of this issue, which is important given its relevance to Community Conversations.

Although all respondents expressed preference for research into causes of childhood mental health problems, a greater proportion of those with lived experience thought it was more important to focus research on the treatment of mental health conditions. This result mirrored findings by Banfield et al [51] who discovered a gap between existing mental health research, which was mainly causal and consumers’ priorities for research, which favoured research into treatments. This seeming tension between the agenda of some researchers versus that of consumers was echoed in a study by O’Donnell et al [52] where research organisations surveyed about consumer involvement in their research expressed concern that consumers might be biased towards applied research at the expense of basic research. The James Lind Alliance in the UK [53] serves as a counter to this attitude, as it was set up in recognition that patient and carer involvement in health treatment research results in solutions that have more practical utility and consequently are far more likely to be successful. The Institute’s Participation Program follows a similar tenet; however, these study findings illustrate the need for balance in seeking the public’s views with respect to designing an equitable research agenda.

In choosing who should have most say in determining research priorities relevant to a health condition, the majority of respondents chose ‘only those with experience of a condition’, as opposed to everyone in the community. Interestingly, there was little difference in this response depending on whether the respondents themselves had a lived experience with a serious health problem (60%) or not (57%).

### Community Conversations

The move to adopt more deliberative processes in a health care setting began in the 1990s out of a desire to involve the public in a two-way interaction with decision makers. [4] However, the Conversations differ from most of the public deliberation processes conducted outside Australia in that all of the participants’ views are captured, without need for justification or in the expectation that a common view or decision must be reached, nevertheless, there are similarities between the two methods. Both processes seek input from members of the community through a facilitation process and views are shared so that parties are exposed to others’ points of view, which may influence how they think and decide on priorities. Similarly, in the eyes of researchers, both processes can suffer from the same perceived lack of legitimacy and generalisability in that they involve relatively small numbers of people. [17] Abelson et al suggest that scaling up exercises may be one solution to increasing legitimacy, particularly if the topic is one likely to impact a large proportion of the population. [31]

Some of the research priorities identified during the Conversations were ones which had not previously emerged through other research processes and this illustrates one of the benefits of this method for seeking consumer involvement. That is, a process where the respondents are not provided with a pre-existing list from which to choose—as in a telephone survey, but rather where they suggest the issues through a facilitated and open discussion. While participants in a deliberative process are more likely to act from an aspect of common concern rather than individual agendas, [47] it is worthwhile noting that the number of attendees to both conversations was very small and it is possible that a different group of attendees with different lived experiences may have resulted in an entirely different list of suggestions and priorities. Nevertheless, the Participation Program is built on the philosophy that all views are equally important and valued, and is in line with an emerging understanding of the value of this type of approach. For example, Beresford dismisses the notion held by some researchers that experiential knowledge can be too subjective or distorting. [48] This is in contrast with those who hold that there is a risk that the views of different minority groups in the community may be overlooked using small samples of participants in deliberative processes. [54]

We found that using the random telephone survey as a means for recruiting participants to a Community Conversation on education proved to be highly ineffective. Twenty people accepted the invitation to attend the Community Conversation, however, only two respondents, plus one partner, attended. While we do not know the reasons for non-attendance, extreme weather conditions on the night may have been a contributory factor. However, even if all of those who accepted the invitation had attended the event, it would still represent a very small fraction of the 816 respondents to the survey.

The 2011 National Survey of Volunteering Issues found that 75% of people preferred regularly volunteering for the same organisation as the most preferred way of volunteering. [41] Asking people to volunteer their time to attend a discussion forum with an organisation with which they are not familiar may have had an impact on their willingness to attend. Reasons given by respondents for not being interested in attending a Community Conversation tended to fall mainly into two categories of ‘not enough time’ and ‘lack of interest’. In comparison, the Community Conversation recruited through the Participation Program’s extensive network resulted in the attendance of 11 people. Although we had aimed at recruiting 20–30 people, this number was higher than for the group recruited through the telephone survey. Given the network is approximately 550 strong, and not everyone would be interested in the topic of childhood education, the higher participation rate demonstrates the benefits of having strong community relationships built through the Participation Program. This is supported by the research of Taras et al who found that community participation in research was enhanced when the research institution built relationships with the community first. [55]

Although there was no intention to compare the two methods used to capture community views on our research program, it is worth observing the differences associated with the methods. The two methodologies, a cross-sectional telephone survey and a public discussion forum (aka Community Conversations), captured different types of input and there is a place for both, depending on budget and anticipated outcomes. Telephone surveys typically are highly structured with pre-existing categories and can cover many subjects [56], while discussion forums or deliberative processes are far more interactive and result in more in-depth exploration of a smaller number of issues. A telephone survey can also explore a subject in depth through the use of open questions, but it can be an expensive and one-sided process. In this study, the telephone survey consisted of mostly closed questions, designed to reduce cost and respondent burden alike, and there was little detailed exploration of a single issue. Nevertheless, the larger sample size enabled useful quantitative analysis. As stated by Johri et al [57] the value of a phone survey needs to be balanced against several shortcomings, which include the lack of time that people are given to consider the issues being canvassed. Although the survey produced useful information about a wide range of community views on child health research, in contrast, the Conversations provided a richer view of a single subject, at a cheaper cost.

The results of this project suggest that using the Participation Program Network can be a useful, low cost method for an Institute researcher to obtain community involvement in child health research. There is nothing precluding the conduct of a Conversation designed to elicit views on a range of subjects, in the same way a telephone survey would, albeit at the cost of analytic capacity. However, this comes with the concomitant need to continue to build strong relationships with the broader community, and to maximise the participation of as diverse a group of people as possible.

## Conclusion

The principles and value of community engagement are widely and well-articulated, and our survey results showed that people think it is very important for the community to have a say in the planning and translation stages of research. Across the world, methodologies for community engagement are still being developed and refined. Many organisations and governments strongly encourage and endorse the concept of community involvement as a natural and necessary part of research, with some providing detailed instructions on how to undertake this type of activity. [6,11,16,27,58,59,60] In Australia, NHMRC has developed a national framework for consumer involvement but this suffers from several barriers to full implementation [15]. Although people see the value of community input to research, clearly their willingness to participate at all levels is not as strong.

This study demonstrated that both of the methodologies employed could contribute to gaining the involvement of community members in research initiatives. However, our findings demonstrated the importance of using quota sampling and including mobile-only households in telephone surveys, if representativeness and diversity of views are valued by the researcher. While the small numbers involved in the Community Conversations limits the ability to draw conclusions beyond the context of this study, we did observe that participants, as well as those merely willing to attend a conversation, not only had a similar demographic profile but they also expressed very similar views to the rest of the sample in the telephone survey. Lastly we noted the importance of establishing relationships with the community in order to convince people to commit a greater amount of time to sharing their views in person. Using a ‘cold calling’ telephone survey approach was clearly an ineffective recruitment method, without having first built a relationship with the respondent.

While community participation in health and medical research has been a priority area for at least a decade, it is possible that many community members in Australia are not aware of all the ways in which they can be involved in, or contribute to, research. More could be done to heighten public awareness about community participation in research, as well as to further understanding within the health and medical research sector about ways in which to benefit from community participation in every phase of the research process. Further studies are also needed to evaluate participatory methods in research and thus assist researchers in understanding the applications of each, and the validity which public involvement can bring to research decisions.

## Supporting Information

S1 Appendix Community Participation in Child Health Research Questionnaire. (PDF)

S2 Appendix Results from Community Conversations. (PDF)
