# Supplementary material for: Two Methods for Engaging with the Community in Setting Priorities for Child Health Research: Who Engages?
Source: PLoS One. 2015 May 4;10(5):e0125969. doi: 10.1371/journal.pone.0125969 (PMC4418596; doi:10.1371/journal.pone.0125969)
Supplement: S1 Appendix — (PDF) [file pone.0125969.s001.pdf]

Telethon Institute for Child Health Research
Community Participation in Child Health Research Questionnaire

Introduction Script

Hello my name is _______________ from [insert field agency name] and I am ringing on behalf of the Telethon Institute for Child Health Research. The Telethon Institute is interviewing members of the community aged over 18 to seek their opinions on aspects of child health research in WA.

I would like to ask you a few questions, which should only take about 10 to 15 minutes to answer. Your participation is completely voluntary and no identifying information will be released in the survey results.

Are you interested in participating?

IF YES, SAY:
We really appreciate you agreeing to participate in the survey. We will be gauging your attitudes and opinions about a number of importance issues relating to child health research. For your reference, federal privacy laws protect the confidentiality of any comments you make in relation to this survey and all responses will be used solely for research purposes.

S1) To start off with, do you or anyone in your household work in or for…

Advertising (including media)	1
Market Research	2
Any medical or health related organisation	3
None	99

S2) Record gender.

Male	1
Female	2

S3) Which age range do you fall into?

Under 18	1
18-24	2
25-34	3
35-44	4
45-54	5
55-64	6
65+	7
Prefer not to say	8

S4) Do you have any children?

Yes	1
No	2

S5) And how many children do you have who are aged less than 18 years?

None	1
1	2
2	3
3	4
4	5
5	6

6	7
7+	8

S6) And how many children (aged under 18?) live at your address at least 50% of the time?

None	1
1	2
2	3
3	4
4	5
5	6
6	7
7+	8

CURRENT RESEARCH PRIORITIES

Q1. We'd like to start by getting an idea of how you rate your interest in the area of child health research. Firstly, using a scale from 0 to 10 where 0 is extremely uninterested and 10 is extremely interested, how much do you feel you know about the area of child health research in general? You can also let me know if you have no opinion on this.

Extremely uninterested										Extremely interested	Don't know	No opinion
0	1	2	3	4	5	6	7	8	9	10	98	99

Q2. And now, using the same scale from 0 to 10 where 0 is extremely uninterested and 10 is extremely interested, how interested are you in providing an opinion on matters relating to child health research in general?

Extremely uninterested										Extremely interested	Don't know
0	1	2	3	4	5	6	7	8	9	10	98

Q3. I'm now going to read out a list of topics relating to areas of research conducted by the Telethon Institute. Firstly, after I have read them all out, I want you to tell me the ONE that you believe is the MOST important area for the Telethon Institute to research. Then once you have chosen the MOST important, I will get you to rate the importance of each of the others on a scale from 0 to 10.

I'll now read the whole list so you can tell me which one you believe is the MOST important for the Telethon Institute to research. Here we go…

a)	Early childhood education (that's kids aged 3-6 years, attending kindy, pre-primary or year one [DO NOT READ OUT FOLLOWING DEFINITION BUT HAVE AVAILABLE IF ASKED FOR MORE INFORMATION] This refers to the formal teaching of young children, usually outside of the home by someone other than their family, most often at a school.	1
b)	Language development [DO NOT READ OUT FOLLOWING DEFINITION BUT HAVE AVAILABLE IF ASKED FOR MORE INFORMATION] This refers to the process people go through to learn how to speak and write.	2
c)	Childhood obesity [DO NOT READ OUT FOLLOWING DEFINITION BUT HAVE AVAILABLE IF ASKED FOR MORE INFORMATION] This refers to a condition where excess body fat negatively affects a child's health or wellbeing.	3
d)	Children's nutrition [DO NOT READ OUT FOLLOWING DEFINITION BUT HAVE AVAILABLE IF ASKED FOR MORE INFORMATION] This refers to the food and drink consumed by children under the age of 18..	4
e)	Children's mental health [DO NOT READ OUT FOLLOWING DEFINITION BUT HAVE AVAILABLE IF ASKED FOR MORE INFORMATION] This refers to the emotional and behavioural problems that children may experience, which can impact on their development and wellbeing. This includes things such as mood disorders.	5
f)	None of the above	98
g)	No opinion	99

Q4. So you've told me **[INSERT ANSWER FROM Q3]** is the most important, how would you rate its importance on a scale from 0 to 10 where 0 is extremely unimportant and 10 is extremely important?

Now, keeping this rating for the most important research area in mind, I'm going to get you to rate the remaining research areas.

IF RESPONDENT GIVES EQUAL OR HIGHER TO THEIR MOST IMPORTANT RESEARCH AREA, ASK THEM TO REASSESS WHICH IS THE MOST IMPORTANT.

		Extremely unimportant										Extremely important	No Opinion
a)	Early childhood education	0	1	2	3	4	5	6	7	8	9	10	99
b)	Language development	0	1	2	3	4	5	6	7	8	9	10	99
c)	Childhood obesity	0	1	2	3	4	5	6	7	8	9	10	99
d)	Children's nutrition	0	1	2	3	4	5	6	7	8	9	10	99
e)	Children's mental health	0	1	2	3	4	5	6	7	8	9	10	99

EDUCATION RESEARCH

Q5. I would now like to ask you some questions about research into childhood education and your possible experience with the school system in WA. To start with, again I'm going to read you a list of areas the Institute could conduct research into and I want you to tell me which ONE you believe is the MOST important.

RANDOMISE ORDER		
a)	The impacts of non-attendance at school due to illness, holidays or for some other reason.	1
b)	How to help children with learning difficulties in school [DO NOT READ OUT FOLLOWING DEFINITION BUT HAVE AVAILABLE IF ASKED FOR MORE INFORMATION] This can be either a disability such as dyslexia or something else that affects how a child learns.	2
c)	How to help children with ADHD in school [DO NOT READ OUT FOLLOWING DEFINITION BUT HAVE AVAILABLE IF ASKED FOR MORE INFORMATION] This is a psychological disorder that is characterised by a persistent pattern of inattention and/or hyperactivity that infers with functioning or development.	3
d)	None of the above	98
e)	No opinion	99

Q6. So you've told me **[INSERT ANSWER FROM Q5]** is the most important, how would you rate its importance on a scale from 0 to 10 where 0 is extremely unimportant and 10 is extremely important?

Now, keeping this rating for the most important research area in mind, I'm going to get you to rate the remaining research areas.

IF RESPONDENT GIVES EQUAL OR HIGHER TO THEIR MOST IMPORTANT RESEARCH AREA, ASK THEM TO REASSESS WHICH IS THE MOST IMPORTANT.

		Extremely unimportant										Extremely important	No opinion
a)	The impacts of non-attendance at school	0	1	2	3	4	5	6	7	8	9	10	99
b)	How to help children with learning difficulties in school	0	1	2	3	4	5	6	7	8	9	10	99
c)	How to help children with ADHD in school	0	1	2	3	4	5	6	7	8	9	10	99

Q7. Do any of your children currently attend primary or secondary school?

Yes	1
No	2

Q8. And what type of school do they currently attend? If you have more than one child in school, you can provide multiple answers.

Government	1
Non-Government	2
Home school	3
Other (please specify)	4

Q9. Have you or any children close to you, ever experienced any learning difficulties at school? Learning difficulties can be either a disability such as dyslexia or something else that affects how a child learns. It can include several areas of functioning which make learning things in the usual manner hard.

Yes	1
No	2
Prefer not to say	3

Q10. Using a scale from 0 to 10 where 0 is poor and 10 is excellent, how would you rate the standard of school education in WA in each of the following environments?

	Poor										Excellent	Can't comment
a) Government primary and secondary schools	0	1	2	3	4	5	6	7	8	9	10	99
b) Non-Government primary and secondary schools	0	1	2	3	4	5	6	7	8	9	10	99

HEALTH RESEARCH

Q11. The next few questions are about areas of the Telethon Institute's research into childhood obesity. Which of the following do you believe should be the MAIN focus of the Institute research activities?

**Identifying the causes** of childhood obesity	1
**Identifying ways to treat** childhood obesity	2
Both equally important	3
No opinion	99

Q12. Again, I'm going to read you a list of potential research areas relating to childhood obesity. First, I'd like you to tell me which ONE you believe is the MOST important area for the Institute to research.

a) Investigating ways to improve children's nutrition	1
b) Investigating ways to increase physical activity in children	2
c) Investigating ways to reduce time children spend in sedentary activities, such as using computers or watching TV	3
d) None of the above	98
e) No opinion	99

Q13. Now, using a scale from 0 to 10 where 0 is extremely unimportant and 10 is extremely important, how do you rate the importance of each of these research topics?

	Extremely unimportant										Extremely important	No opinion
a) Investigating ways to improve children's nutrition	0	1	2	3	4	5	6	7	8	9	10	99
b) Investigating ways to increase physical activity in children	0	1	2	3	4	5	6	7	8	9	10	99
c) Investigating ways to reduce time children spend in sedentary activities, such as using	0	1	2	3	4	5	6	7	8	9	10	99

computers or watching TV

MENTAL HEALTH RESEARCH

Q14. I would now like to ask you some questions about mental health research. Which do you think is more important for the Institute to research – the CAUSES of mental health problems in children or how to best TREAT mental health problems once they have occurred? So your options are:

Causes	1
Treatment	2
Equally important	3
Neither	4
No opinion	99

Q15. Have you or has anyone you know well ever been diagnosed with a mental health problem?

Yes	1
No	2
Prefer not to say	3

Q16. Have you or has anyone you know well ever had any experience with mental health services in WA?

Yes	1
No	2
Prefer not to say	3

Q17. And, using a scale from 0 to 10 where 0 is poor and 10 is excellent, how would you rate the standard of mental health care in WA?

Poor										Excellent	Don't know
0	1	2	3	4	5	6	7	8	9	10	98

COMMUNITY PARTICIPATION

The next few questions are about the community working together with researchers to make decisions about their research.

Q18. On a scale from 0 to 10 where 0 is extremely unimportant and 10 is extremely important, how important do you think it is for the community to have a say in setting the Institute's research priorities – that is deciding what areas the Institute researches?

Extremely unimportant										Extremely important	No opinion
0	1	2	3	4	5	6	7	8	9	10	99

Q19. Using that same scale, how important is it for the community to have a say in each of the following stages of the Institute's research activities?

	Extremely unimportant										Extremely important	No opinion
a) Deciding what are the most important things to research i.e. setting research priorities	0	1	2	3	4	5	6	7	8	9	10	99
b) Planning the research i.e. deciding how to conduct the research	0	1	2	3	4	5	6	7	8	9	10	99
c) Deciding how to use or act on the results of research projects (e.g. whether the government should take action)	0	1	2	3	4	5	6	7	8	9	10	99

Q20. I'm now going to read out three groups and I want you to rank them from 1 (being most important) to 3 (being the least important) based on who you believe should have the MOST say in deciding what research projects are undertaken by the Institute? Here's the three groups: RO. Now which do you believe should be ranked first as most important? And second? And third as the least important? SR

Government	1	2	3
Members of the community	1	2	3
Institute researchers	1	2	3

Q21. When the Institute researches a particular condition, do you think EVERYONE in the community should have a say in planning the research or ONLY THOSE PEOPLE WHO HAVE EXPERIENCE of the condition? So your choices are…

Everyone	1
Only those who experience the condition	2
Both	3
Neither	4
Don't know	98
No opinion	99

Q22. Using a scale from 0 to 10 where 0 is extremely ineffective and 10 is extremely effective, how effective do you think each of the following methods could be for the community to help shape decisions regarding the Institute's research?

RANDOMISE ORDER	Extremely ineffective									Extremely effective	No opinion
a) Participating in surveys about research	0	1	2	3	4	5	6	7	8	9	10	99
b) Participating in focus groups or workshops	0	1	2	3	4	5	6	7	8	9	10	99
c) Participating in training in how to partner with researchers	0	1	2	3	4	5	6	7	8	9	10	99
d) Having a community representative on advisory groups	0	1	2	3	4	5	6	7	8	9	10	99
e) Donating money to specific areas of research	0	1	2	3	4	5	6	7	8	9	10	99

Q23. Using a scale from 0 to 10 where 0 is extremely uninterested and 10 is extremely interested, how interested are you in keeping up to date with what is happening in child health research?

Extremely uninterested										Extremely interested	No opinion
0	1	2	3	4	5	6	7	8	9	10	99

Q24. Have you seen or heard any information or advertising about child health research in any of the following?

Newspaper – Sunday Times or West Australian	1
Newspaper - community	2
Television	3
Radio	4
Social media such as Facebook or Twitter	5
Online via websites (Please specify)	6
Via email	7
Health professionals / clinics	8
Special interest group	9
Word of mouth	10
Other (please specify) DNRO	11

Q25. And what is your most preferred method of communication for obtaining information about child health research? SR

Newspaper – Sunday Times or West Australian	1
Newspaper - community	2
Television	3
Radio	4
Social media such as Facebook or Twitter	5
Online via websites (Please specify)	6
Via email	7
Health professionals / clinics	8
Special interest group	9
Word of mouth	10
Other (please specify)	11

Q26. From time to time the Telethon Institute conducts interactive workshops, called Community Conversations, to discuss specific aspects of child health research in more detail. There is a short presentation on the topic and then small group discussions about specific questions. The process is interactive and promotes people being able to have a say about the issue. If you attend one of these events, the Institute offers payment for out-of-pocket expenses such as travel and parking up to the value of $30. Would you be interested in attending a workshop like this?

Yes	1
No	2
Don't know	3

Q27. And which of the following times would be the most convenient for you to attend an event?

Weekday during the day	1
Weekday during the evening	2
Weekend during the day	3

Q28. Occasionally the Institute calls for volunteers to take part in studies or trials, such as testing a new vaccine or studies into various health conditions. Are you open to the idea of taking part in this type of study?

Yes	1
No	2
Maybe - would need more information	3
Don't know	4

Q29. Occasionally the Institute calls for volunteers to take part in studies or trials, such as testing a new vaccine or studies into various health conditions. Are you open to the idea of taking part or letting your child take part in this type of study?

Yes	1
No	2
Maybe - would need more information	3
Don't know	4

Q30. Would you like to register to receive information regarding opportunities to participate in research?

Yes [Record NAME, EMAIL ADDRESS and PHONE NUMBER to add to TICHR database]	1
No	2

We're nearly at the end of our survey. The last few questions will help us understand you a little more and ensure we speak to a broad cross-section of the community.

Q31. Have you done any of the following in the past 12 months?

Participated in a consumer advocacy or special interest action	1

group	
Participated in your local school P&C	2
Attended a community forum covering local issues	3
Volunteered your time or services for a group or organisation	4
Sought signatures for a petition	5
Written an opinion letter to a newspaper	6
Any other community-based activity (please specify)	7

Q32. Do you or does any member of your immediate family have a serious health problem (for example, a problem that would require them to see a doctor regularly or to spend time in hospital)?

Yes	1
No	2
Prefer not to say	3

Q33. Using a scale from 0 to 10 where 0 is strongly disagree and 10 is strongly agree, to what extent do you agree with the following statements?

	Strongly disagree										Strongly agree
a) It should be compulsory for all children to be vaccinated	0	1	2	3	4	5	6	7	8	9	10
b) The legal drinking age for alcohol should be raised to 21	0	1	2	3	4	5	6	7	8	9	10
c) Unhealthy food should have a tax imposed on it to discourage people eating it	0	1	2	3	4	5	6	7	8	9	10

Q34. Are you…?

Single	1
Married	2
In a de facto relationship	3
Separated	4
Divorced	5
Widowed	6
Other (please specify)	7
Prefer not to say	98

Q35. What is the highest level of education you have completed?

Tertiary qualification	1
Year 11-12	2
Year 8-10	3
No formal education(did not go to school)	4
Prefer not to say	5

Q36. Are you currently in paid employment?

Yes	1
No	2
Prefer not to say	3

Q37. In the last week, how many hours did you work in paid employment?

1-9	1
10-19	2
20-29	3
30-39	4
40-49	5
50-59	6

60-69	7
70-79	8
80-89	9
90-99	10
100+	11

Q38. What is your combined annual HOUSEHOLD income before tax?

No income	1
Less than $32,000 per year ($1-$615 per week)	2
$32,000 to less than $65,000 per year ($616-$1,249 per week)	3
$65,000 to less than $130,000 per year ($1250-$2,499 per week)	4
$130,000 to less than$210,000 per year ($2500-$4038 per week)	5
$210,000 or more per year ($4,039 or more per week)	6
Prefer not to say	7

Q39. What country were you born in?

Australia	1
Canada	2
China	3
Hong Kong	4
India	5
Indonesia	6
Japan	7
Malaysia	8
New Zealand	9
Republic of Korea	10
Singapore	11
Sri Lanka	12
Thailand	13
United Kingdom (including England, Ireland, Scotland and Wales)	14
United States of America	15
South Africa	16
Italy	17
Greece	18
Vietnam	19
Germany	20
Other (please specify)	21
Prefer not to say	23

Q40. What is your postcode?

Q41. Thanks very much, that is the end of the formal part of the survey. There's just one more thing I want to ask you about. Earlier you said that you would be interested in attending a workshop on child health research. At 7.00pm on Wednesday 28th August, the Telethon Institute will be conducting a workshop on childhood education. The workshop will run for two hours and will be held at the Institute's premises in Subiaco. Would you be interested in attending?

Yes	1
No	2
Not sure	3

Q42. Great, may I have your contact details for the invitation please?

Name:
Contact number:
Address: [If would prefer posted invite]
Email: [If would prefer mailed invite]

END

Thank you very much for your participation, it is greatly appreciated. Should you have any queries regarding this survey, you can contact Sally Braidwood at Painted Dog Research on 08 9227 6464. If you wish to make a complaint about the project you can contact the Human Research Ethics Office at the University of Western Australia on 6488 1610.

Thank you again for taking the time to complete this survey – we really appreciate it.
