# Supplementary material for: Two Methods for Engaging with the Community in Setting Priorities for Child Health Research: Who Engages?
Source: PLoS One. 2015 May 4;10(5):e0125969. doi: 10.1371/journal.pone.0125969 (PMC4418596; doi:10.1371/journal.pone.0125969)
Supplement: S2 Appendix — (PDF) [file pone.0125969.s002.pdf]

Appendix 2

Results from Community Conversations

Topic – Student Attendance and Educational Outcomes: Every Day Counts

Questions

1. What is your reaction to these results? How do you think the results might influence parents' attitudes and behaviour towards school attendance?
2. What do you think can be done to improve attendance at school?
3. Where do you currently get information about issues to do with education? What are your suggestions for making this better?
4. What should be the priority areas for education-related research?

Results

Within the theme of culture and language issues raised included the development of programs where children have the opportunity to be taught in their own language, especially in remote Indigenous communities; the development of transition programs to mainstream education for refugee children; and examining Indigenous education and its impact on the 'whole village'.

With regard to bullying, attendees considered the effects of different approaches to bullying between schools and educational systems, specifically authoritative approaches versus the current 'no blame' policy. They stated that there needs to be consistency between, and standardisation of, these approaches.

Community members expressed the view that awareness about education should start much earlier than formal school programs and discussed the influence that mothers groups, prominent local groups and the '*Communities that Care*' program could have on this process.

They also addressed the issue of schools becoming more strongly linked to the community by being seen as an integral part of the community. Therefore, broader holistic approaches to educational issues

were suggested to explore ways to include the whole community, so that children and their families are not made to feel isolated or different.
